# Factors associated with suicidal ideation in junior high school students with autism spectrum disorder in Japan: A cross‐sectional observational study

**DOI:** 10.1002/pcn5.70272

**Published:** 2026-01-05

**Authors:** Yu Matsumoto, Kentaro Kawabe, Fumie Horiuchi, Toshihiro Jogamoto, Rie Hosokawa, Kiwamu Nakachi, Junya Soga, Saori Inoue, Maya Kusunoki, Mariko Eguchi, Shu‐ichi Ueno

**Affiliations:** ^1^ Department of Neuropsychiatry Ehime University Graduate School of Medicine Toon City Japan; ^2^ Department of Child Psychiatry Ehime University Graduate School of Medicine Toon City Japan; ^3^ Department of Pediatrics Ehime University Graduate School of Medicine Toon City Japan

**Keywords:** Autism Screening Questionnaire, autism spectrum disorder, General Health Questionnaire, junior high school students, suicidal ideation

## Abstract

**Aim:**

Suicide is a leading cause of death among adolescents. Several studies have reported higher suicidal ideation (SI) rates in individuals with autism spectrum disorder (ASD) than in those without ASD; however, risk factors for SI remain unclear, especially among adolescents. This study aimed to investigate the factors contributing to SI among junior high school students with ASD in Japan.

**Methods:**

We conducted a cross‐sectional observational study of junior high school students who visited the Center for Child Health, Behavior, and Development, Ehime University Hospital. Medical records from April 2015 to March 2022 were examined. Participants completed the Strengths and Difficulties Questionnaire (SDQ) and General Health Questionnaire 30 (GHQ30), while their parents completed the Autism Screening Questionnaire, Attention Deficit Hyperactivity Disorder Rating Scale, and Social Responsiveness Scale. SI was assessed using item 28 of the GHQ30: “make away with yourself.” Multiple logistic regression analysis was performed with SI as the dependent variable.

**Results:**

Participants were categorized into ASD (*n* = 84) and non‐ASD (*n* = 166) groups. The prevalence of SI was similar in both groups (*p* = 0.478). In the ASD group, multiple logistic regression analysis revealed that the SDQ subscales “Peer Problems” and “Emotional Symptoms” were significantly associated with SI (odds ratio [OR]: 1.63, 95% confidence interval [CI]: 1.22–2.19, OR: 1.44, 95% CI: 1.14–1.83).

**Conclusion:**

Approximately 40% of junior high school psychiatric outpatients had SI, irrespective of autistic tendencies. Our study suggests the importance of enhancing peer connectedness among students with ASD who experience SI.

## INTRODUCTION

Suicide, the act of intentionally taking one's own life, is a leading cause of premature death. According to the World Health Organization (WHO) database, 727,000 people died by suicide in 2021.^1^ The total number of global suicide deaths increased by 6.7% (95% uncertainty interval: 0.4–15.6) between 1990 and 2016.^2^ The WHO Comprehensive Mental Health Action Plan (2013–2030) aims to reduce the global suicide rate by one‐third between 2013 and 2030.^3^ In Japan, suicide rates markedly increased in 1998, making it a major social concern. Following approval of the Basic Act on Suicide Prevention in 2006, the “General Policies of Comprehensive Measures against Suicide” was formulated in 2007 by the government to serve as official guidelines for suicide prevention measures.^4^ The general policies provided for measures against suicide were strongly promoted through close cooperation between the government, local governments, medical institutions, and public organizations. This joint effort aimed to reduce suicide death rates by ≥20% between 2005 and 2016.^4^ Despite an overall decline in suicide rates across Japan in recent years, suicide rates in adolescents have markedly increased.^5^, ^6^

According to the WHO database of causes of death in 2021, suicide is the leading cause of death among teenagers in Japan, with a 7.0% suicide rate.^1^ Notably, Japan is the only country among the Group of Seven (G7) with such ratings. Since 2017, the government has prioritized suicide prevention strategies among adolescents.^4^ Suicide in adolescents is an urgent issue; however, the causes of suicide are variable and complex. Factors may include school‐related issues (such as school refusal, poor school performance, and friendships), family background (such as parental discord), financial difficulties, and personal challenges (such as one's own character and mental or personality disorders).^7^ Suicidal ideation (SI) is a well‐known risk factor of suicide,^8^ with an approximate global lifetime prevalence rate of 9.2%.^9^ According to systematic reviews of young children, SI rates were 7.5% in children aged 6–12 years and 15.1% in pre‐adolescents.^5^, ^10^ SI is caused by various risk factors, including biological, psychological, and socioenvironmental factors. SI varies from non‐specific to specific thoughts on death and suicide plans.^1^ Non‐specific SI includes passive suicidal thoughts, such as the belief that life is not worth living or wishing to no longer be alive.^11^, ^12^, ^13^ Children typically begin to grasp the concept of death between ages 6 and 10, and this understanding becomes temporarily “fuzzy” again during adolescence.^14^ Adolescents often express fuzzy SI using vague phrases such as “to make away with self.” The wording of questions on SI should be tailored to the child's age and cognitive and emotional functioning, as directly using the word “suicide” may not be appropriate for children.^12^ These findings highlight the need for developmentally sensitive approaches to assessing SI in adolescents, as conventional terms such as “suicide” may fail to capture their distress.

Autism spectrum disorder (ASD) is a psychiatric disorder associated with SI.^15^, ^16^, ^17^ ASD is a neurodevelopmental disorder characterized by distinct social communication and interaction styles, varied sensory processing sensitivities, deep interest, and preferences for predictability and routines.^18^ Several studies have suggested that suicide and SI rates are higher among individuals with ASD than those without ASD.^15^, ^16^, ^17^ Systematic reviews have reported SI rates of 20%–66% among individuals with ASD, and individuals with ASD accounted for 7.3%–15% of the suicidal population.^19^, ^20^, ^21^ Several risk factors for suicidality have been identified, including peer victimization, behavioral problems, being Black or Hispanic, being male, lower socioeconomic status, lower education levels, and having a history of self‐harm and depression.^20^, ^21^ Before 2022, only one systematic review and meta‐analysis had reported that approximately 25% of youths with ASD aged < 25 years experienced SI.^22^ Although the review identified some predictors of SI, the specific factors associated with SI in school‐aged adolescents with ASD remain unclear.^22^ Storch et al. reported that approximately 11% of 102 children and adolescents with ASD (aged 7–16 years) experienced SI.^23^ However, the study was limited to participants with anxiety issues, making it insufficient for broader clinical use.^23^ This study is based on the hypothesis that SI is higher in adolescents with ASD than in adolescents without ASD in a clinical setting. Primarily, this study aimed to determine whether adolescents with ASD have higher SI than adolescents without ASD. We also sought to identify the factors associated with SI in adolescents with ASD.

## METHODS

### Participants

This was a cross‐sectional observational study of medical records between April 2015 and March 2022 at the Center for Child Health, Behavior, and Development, Ehime University Hospital. The participants were junior high school students aged 12–15 years who visited the center for the first time. Inclusion criteria were patients with mental illnesses diagnosed by a psychiatrist according to the Diagnostic & Statistical Manual of Mental Disorders, 5th edition (DSM‐5), and requiring continuous outpatient treatment. Exclusion criteria were patients with intellectual disability (intelligence quotient, IQ < 70) and endogenous psychiatric disorders such as schizophrenia and bipolar disorder. We excluded participants who failed to distribute or collect questionnaires, as well as those who submitted an incomplete questionnaire. This study was approved by the Institutional Review Board of the Ehime University Graduate School of Medicine (IRB No. 1607010). The study protocol was explained to participants and their parents. Written informed consent was obtained from all students and their parents.

### Procedures and instruments

Participants were given questionnaires during their first visit to the center. The questionnaires were collected during their second or subsequent visits. For junior high school students, self‐rated versions of the Strengths and Difficulties Questionnaire (SDQ) and the 30‐item General Health Questionnaire (GHQ30) were prepared. For their parents, the Autism Screening Questionnaire (ASQ), Attention Deficit Hyperactivity Disorder Rating Scale (ADHD‐RS‐IV), and Social Responsiveness Scale (SRS‐2) were prepared. Each scale was selected based on the influence of developmental characteristics, interpersonal skills, emotions, and behaviors.

### Self‐rated SDQ

The self‐rated SDQ comprises 25 items and uses a five‐factor scale.^24^ This scale assesses four difficulties: emotional symptoms (ES), conduct problems (CP), hyperactivity–inattention (HI), peer problems (PP), and one strength: prosocial behavior (PB). Each item is assigned a score from 0 to 2, with a total score from 0 to 10 for each subscale. For the difficulties subscale, higher scores indicated greater difficulty. Conversely, for the prosocial behavior subscale as a strength, higher scores indicated more prosocial behavior. The Japanese version of the SDQ was as reliable and useful as the original English version.^25^

### ASQ

Berument et al. (1999) developed the ASQ, which comprises 39 questions on three basic autism disorders and is an effective screening questionnaire.^26^ The Japanese version of the ASQ, standardized by Dairoku, has demonstrated some accuracy as a screening tool.^27^ The cutoff value was set at 13 points, making it an effective screening test.

### ADHD‐RS‐IV

Based on the ADHD criteria of the DSM‐IV, the ADHD‐RS‐IV comprises two subscales to measure the two major ADHD characteristics: inattentiveness (9 items) and hyperactive‐impulsiveness (9 items). Both school and home forms of the ADHD‐RS‐IV have been confirmed to have sufficient reliability and validity.^28^ In this study, the home form of the ADHD‐RS‐IV was selected. Each item is rated by the parents on a scale of 0–3. The highest score indicates the highest severity of ADHD tendency. The Japanese version of the ADHD‐RS‐IV was translated by DuPaul et al. (1998).^28^ The Japanese version of the home form of the ADHD‐RS‐IV was developed with good reliability and validity.^29^

### SRS‐2

The SRS‐2 comprises 65 items assessing autistic traits in individuals aged 4–18 years, and can be completed by parents or teachers.^30^ Each item is scored from 0 to 3, and the total score ranges from 0 to 195, with higher scores indicating a higher degree of social impairment. We selected the parental version for this study. The Japanese version of the parent SRS‐2 is a reliable and valid assessment tool for Japanese children.^31^

### Assessment of SI by GHQ30

The presence or absence of SI was assessed using item No.28 of the GHQ30: “make away with yourself.” The GHQ30 comprises 30 items, which assess general psychopathology and measure mental state.^32^ Respondents were asked to rate each item on four levels according to the degree to which it was applicable during the preceding 2 or 3 weeks. Two scoring systems were used: a Likert scale (0–3) and a two‐point scale (0 or 1). We selected the two‐point scale for this study. The Japanese translation of the GHQ30 has also been validated.^33^ In this study, “definitely not” and “I don′t think so” were scored 0 (indicating no SI), and “has crossed my mind” and “definitely have” were scored 1 (indicating SI).

### Statistical analysis (Studies 1 and 2)

**Figure 1:**
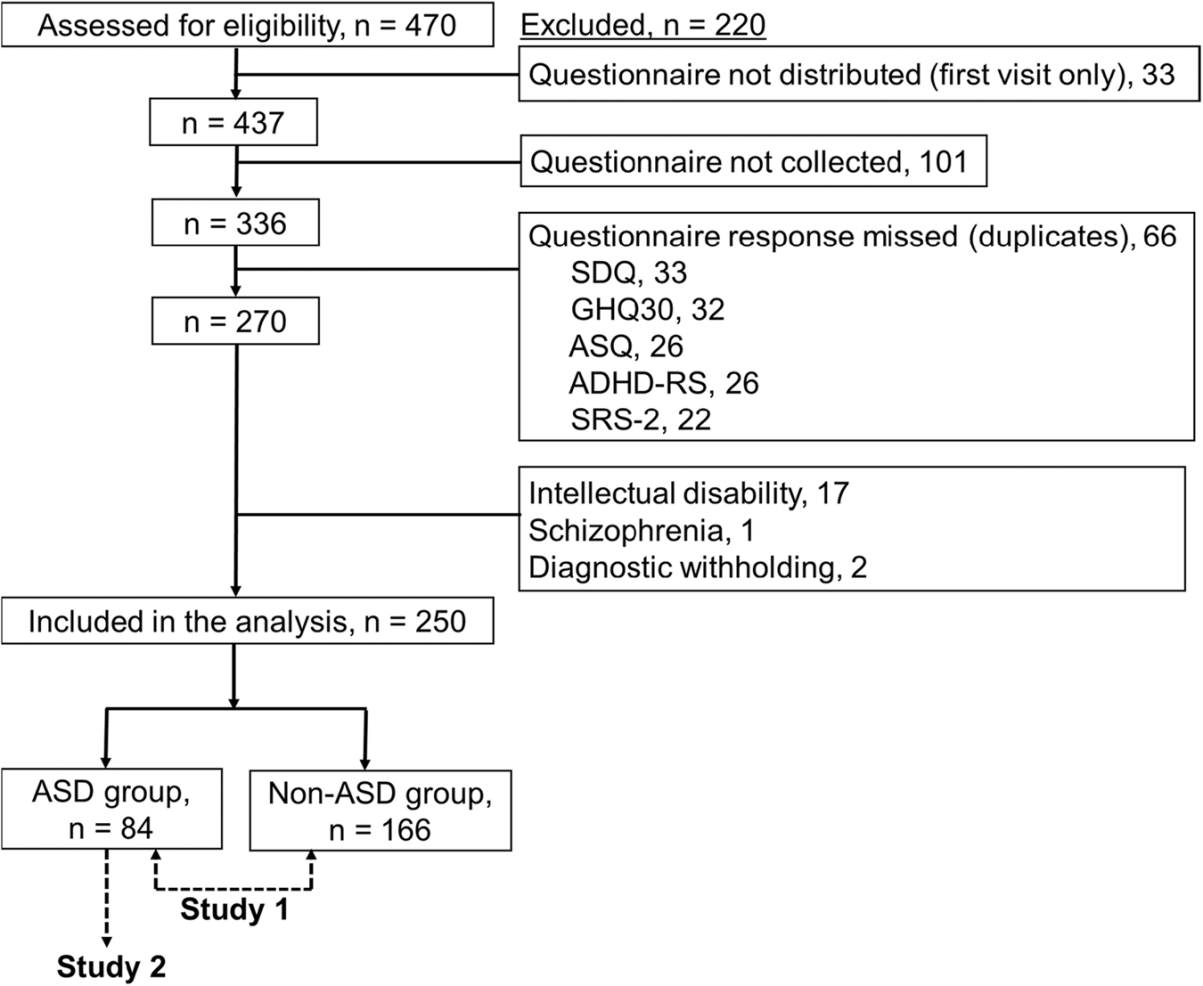
Flow diagram of participants. A total of 250 participants were included in the analysis. Study 1: compare suicidal ideation (SI) between the autism spectrum disorder (ASD) and non‐ASD groups. Study 2: examine factors associated with SI in the ASD group. ADHD‐RS, Attention Deficit Hyperactivity Disorder Rating Scale; ASQ, Autism Screening Questionnaire; GHQ30, 30‐item General Health Questionnaire; SDQ, Strengths and Difficulties Questionnaire; SRS‐2, Social Responsiveness Scale.

This study comprised two parts: Study 1 and Study 2. The participants were categorized into ASD and non‐ASD groups (Figure 1). Study 1 aimed to compare SI, demographic characteristics, and clinical variables between the two groups. The chi‐squared and Mann–Whitney's *U* tests were used for categorical and continuous variables, respectively. Study 2 investigated factors associated with SI within the ASD group. A stepwise multiple logistic regression analysis was performed on the ASD group, with the presence of SI as the dependent variable and each questionnaire score as an independent variable (age, sex, the presence/absence of psychiatric comorbidities, ASQ, ADHD‐RS‐IV, SRS‐2, and the five SDQ subscales). The significance level was set at *p* < 0.05. All statistical tests were performed using the IBM spss Statistics version 26 (IBM Corp., Armonk, NY, USA).

## RESULTS

### Participant characteristics

Overall, 470 participants met the eligibility criteria. Among those participants, 33 did not distribute the questionnaires, and 101 did not collect them, resulting in a collection rate of 76.9% (336 of 470; Figure 1). In addition, 66 participants were excluded due to incomplete responses, and 20 others were excluded due to psychiatric diagnoses that met the exclusion criteria. As a result, a total of 250 participants were included in the final analysis (115 males [46.0%]; age, 13.8 ± 0.84 years). Demographic data and diagnoses of the ASD and non‐ASD groups are presented in Table 1. In the ASD group, the diagnoses were adjustment disorders (*n* = 35, 41.7%), followed by ADHD (*n* = 14, 16.7%), while 34.5% (*n* = 29) had no diagnosis other than ASD. In the non‐ASD group, the diagnoses were adjustment disorders (*n* = 94, 56.6%), followed by feeding and eating disorders (*n* = 11, 6.6%), and somatic symptom disorders (*n* = 10, 6.0%).

**Table 1:** Demographic data and diagnoses of the participants.

	Total (*n* = 250)	ASD (*n* = 84)	Non‐ASD (*n* = 166)	Statistics	*p* value
Age	13.8 ± 0.84	13.7 ± 0.8	13.9 ± 0.8	*t* = −1.319	0.187
Sex (male), *n* (%)	115 (46.0)	55 (65.5)	60 (36.1)	*X* ^2^ = 19.318	<0.001**
*Diagnoses, n (%)*					
Adjustment disorders	129 (51.6)	35 (41.7)	94 (56.6)		
ADHD	21 (8.4)	14 (16.7)	7 (4.2)		
Feeding and eating disorders	13 (5.2)	2 (2.4)	11 (6.6)		
Somatic symptom disorder	11 (4.4)	1 (1.2)	10 (6.0)		
Dissociative disorders	7 (2.8)	1 (1.2)	6 (3.6)		
Conversion disorder	7 (2.8)	0 (0.0)	7 (4.2)		
Tic disorders	6 (2.4)	2 (2.4)	4 (2.4)		
Sleep–wake disorders	6 (2.4)	3 (3.6)	3 (1.8)		
Generalized anxiety disorder	5 (2.0)	0 (0.0)	5 (3.0)		
Major depressive disorder	4 (1.6)	0 (0.0)	4 (2.4)		
Obsessive–compulsive disorder	4 (1.6)	1 (1.2)	3 (1.8)		
LD	3 (1.2)	1 (1.2)	2 (1.2)		
Panic disorder	3 (1.2)	1 (1.2)	2 (1.2)		
Trichotillomania	3 (1.2)	0 (0.0)	3 (1.8)		
Posttraumatic stress disorder	0 (0.0)	0 (0.0)	2 (1.2)		
Selective mutism	0 (0.0)	0 (0.0)	1 (0.6)		
Excoriation	0 (0.0)	0 (0.0)	1 (0.6)		
Others	0 (0.0)	0 (0.0)	3 (1.8)		
Only ASD	29 (11.6)	29 (34.5)	0 (0.0)		

*Note*: Data are presented as *n* (%) of participants or as mean ± SD. Participants were diagnosed by a psychiatrist according to the DSM‐5. Diagnoses indicated as coexistence.

Abbreviations: ADHD, attention deficit hyperactivity disorder; ASD, autism spectrum disorder; DSM‐5, Diagnostic & Statistical Manual of Mental Disorders, 5th edition; LD, learning disability; *n*, number; SD, standard deviation.

* *p* < 0.05;

** *p* < 0.01.

### Comparison of scores between ASD and non‐ASD groups (Study 1)

Between the two groups, sex, SDQ‐PB, ADHD‐RS‐IV, ASQ, and SRS‐2 scores were significantly different (all *p* < 0.001; Tables 1 and 2). Although the prevalence of SI was 41.2% (95% confidence interval [CI]: 35.0–47.6) in all participants, no differences were observed between the groups: 38.1% in the ASD group (95% CI: 27.7–49.3) and 42.8% in the non‐ASD group (95% CI: 35.1–50.7; *p* = 0.478; Table 2). Among 29 participants with ASD without comorbidities, the prevalence of SI was 27.6% (95% CI: 12.7–47.2).

**Table 2:** Comparison of clinical scale scores between autism spectrum disorder (ASD) and non‐ASD groups.

	Total	ASD (*n* = 84)	Non‐ASD (*n* = 166)	Statistics	*p* value
*Self‐reported*					
SDQ‐ES	5.0 ± 2.8	4.6 ± 2.7	5.2 ± 2.8	*t* = −1.793	0.073
SDQ‐CP	2.5 ± 1.8	2.8 ± 1.8	2.3 ± 1.7	*t* = −1.760	0.078
SDQ‐HI	4.5 ± 2.5	4.9 ± 2.3	4.2 ± 2.6	*t* = −1.932	0.053
SDQ‐PP	3.4 ± 2.2	3.8 ± 2.3	3.2 ± 2.1	*t* = −1.896	0.058
SDQ‐PB	5.6 ± 2.2	4.9 ± 1.9	5.9 ± 2.3	*t* = −3.257	0.001**
Suicidal ideation, *n*	103 (41.2)	32 (38.1)	71 (42.8)	*X* ^2^ = 0.503	0.47
*Parents reported*					
ADHD‐RS‐IV	10.7 ± 9.3	15.2 ± 10.5	8.4 ± 7.8	*t* = −4.947	<0.001**
ASQ	5.2 ± 5.2	8.3 ± 6.4	3.6 ± 3.5	*t* = −6.186	<0.001**
SRS‐2	51.8 ± 24.4	67.2 ± 26.3	44.0 ± 19.3	*t* = −6.696	<0.001**

*Note*: Data are presented as *n* (%) of participants or as mean ± SD. Suicidal ideation was defined based on item 28 of the General Health Questionnaire 30 (GHQ30).

Abbreviations: ADHD‐RS‐IV, Attention Deficit Hyperactivity Disorder Rating Scale; ASQ, Autism Screening Questionnaire; *n*, number; SDQ‐CP, Strengths and Difficulties Questionnaire‐conduct problems; SDQ‐ES, Strengths and Difficulties Questionnaire‐emotional symptoms; SDQ‐HI, Strengths and Difficulties Questionnaire‐hyperactivity–inattention; SDQ‐PB, Strengths and Difficulties Questionnaire‐prosocial behavior; SDQ‐PP, Strengths and Difficulties Questionnaire‐peer problems; SRS‐2, Social Responsiveness Scale.

*p < 0.05; **p < 0.01.

### Stepwise multiple logistic regression analysis in the ASD group (Study 2)

In the ASD group, stepwise multiple logistic regression analysis revealed that SDQ‐PP and SDQ‐ES were significantly associated with SI (odds ratio [OR]: 1.63, 95% CI: 1.22–2.19; OR: 1.44, 95% CI: 1.14–1.83; Table 3). However, ASQ, SRS‐2, and ADHD‐RS‐IV scores were not associated with SI. Furthermore, the presence or absence of psychiatric comorbidities was not significantly associated with SI.

**Table 3:** Stepwise logistic regression analysis with suicidal ideation as the dependent variable.

	Single regression			Multiple regression		
	OR	95% CI	Significant probability	OR	95% CI	Significant probability
Age	1.05	0.61–1.80	0.87	‐	‐	‐
Sex	0.52	0.21–1.31	0.17	‐	‐	‐
Presence/absence of psychiatric comorbidities	2.03	0.77–5.38	0.15	‐	‐	‐
*Self‐reported*						
SDQ‐ES	1.56	1.25–1.95	<0.001**	1.44	1.14–1.83	0.002**
SDQ‐CP	1.03	0.80–1.32	0.80	‐	‐	‐
SDQ‐HI	1.07	0.89–1.30	0.47	‐	‐	‐
SDQ‐PP	1.76	1.34–2.30	<0.001**	1.63	1.22–2.19	0.001**
SDQ‐PB	1.09	0.87–1.37	0.47	‐	‐	‐
*Parents reported*						
ADHD‐RS‐IV	0.99	0.95–1.03	0.52	‐	‐	‐
ASQ	0.97	0.90–1.04	0.34	‐	‐	‐
SRS‐2	1.00	0.99–1.01	0.79	‐	‐	‐

*Note*: The dependent variable was the presence of suicidal ideation.

Abbreviations: ADHD‐RS‐IV, Attention Deficit Hyperactivity Disorder Rating Scale; ASQ, Autism Screening Questionnaire; CI, confidence interval; OR, odds ratio; SDQ‐CP, Strengths and Difficulties Questionnaire‐conduct problems; SDQ‐ES, Strengths and Difficulties Questionnaire‐emotional symptoms; SDQ‐HI, Strengths and Difficulties Questionnaire‐hyperactivity–inattention; SDQ‐PB, Strengths and Difficulties Questionnaire‐prosocial behavior; SDQ‐PP, Strengths and Difficulties Questionnaire‐peer problems; SRS‐2, Social Responsiveness Scale.

*p < 0.05; **p < 0.01.

## DISCUSSION

This study examined the prevalence of SI among junior high school students at a child and adolescent psychiatric center and also investigated the factors associated with SI among junior high school students with ASD. To the best of our knowledge, this is the first study to examine SI in junior high school students with ASD in a clinical setting.

According to our previous study, the prevalence of SI was 10.3% (95% CI: 5.9–14.6) among Japanese junior high school students in the general population.^34^ In this study, the prevalence of SI among junior high school students in a clinical setting was 41.2%. According to the Global School‐based Student Health Survey (GSHS) by WHO, comprising 90 countries, the prevalence of SI differs across countries.^35^ Notably, among adolescents aged 13–15 years, the prevalence of SI was lowest in Myanmar (girls: 0.8 ± 0.5%; boys: 0.7 ± 0.7%). However, the highest prevalence of SI among boys and girls (aged 13–15 years) was in Samoa (37.1%, 95% CI: 33.8–40.5) and Kiribati (36.2%, 95% CI: 31.9–41.7), respectively. This finding suggests that SI among junior high school students is higher in Oceania Island countries than in other countries. In China, the only East Asian country participating in the GSHS, the prevalence of SI was 22.9% (21.7–24.0, *p* < 0.001) among middle school students.^36^ To date, no clinical data on the prevalence of SI among junior high school students exist.

The prevalence of SI in this study was considerably higher than that in the general junior high school population. Contrary to our initial hypothesis, no significant difference was observed in the prevalence of SI between students with and without ASD (approximately 40%). One possible explanation is that all participants were psychiatric outpatients referred to a specialized center and may have had elevated psychological distress regardless of ASD diagnosis. Although all questionnaires were administered during the first visit, the fact that participants had already been referred for psychiatric evaluation suggests a preexisting level of symptom severity. Therefore, the high prevalence of SI may reflect the clinical characteristics of the sample rather than diagnostic group differences. SI is a recognized symptom of a broad spectrum of psychiatric disorders, including schizophrenia, major depressive disorder, obsessive–compulsive disorder, substance use disorder, sleep disturbances, and alcohol‐related disorders.^37^, ^38^, ^39^, ^40^, ^41^, ^42^ Furthermore, the prevalence of SI was 27.6% in participants with ASD with no comorbid psychiatric disorders in this study. This finding suggests that adolescents with ASD may have a higher tendency to experience SI than those without ASD. Furthermore, 50%–60% of individuals experiencing SI do not disclose them to others.^43^ Thus, the prevalence of SI in this study may be an underestimation of its actual occurrence.

Our findings indicated that peer relationship difficulties and emotional symptoms are significantly associated with SI among adolescents with ASD. Moreover, a characteristic of ASD is difficulty in understanding the concept of friendship.^44^, ^45^ To date, only one systematic review on the nature of friendship in adolescents with ASD is available.^46^ This review reported that adolescents with ASD have fewer friends, less friendship reciprocation, and lower friendship quality and contact frequency. Although most adolescents with ASD had fewer friends than their peers, they were satisfied with their friendships.^47^ In early elementary school, students with ASD usually have similar numbers of reciprocal friendships with their peers; however, the disparity widens in later elementary grades.^48^ The transition to junior high school requires the rebuilding of friendships and is therefore a stressful period of social adaptation.^49^ Although most adolescents with ASD desire meaningful friendships, their desires are often unmet, resulting in feelings of loneliness.^50^, ^51^ Recently, the incidence of school refusal has been increasing annually among junior high school students in Japan.^52^ One contributing factor to school refusal is a lack of quality friendships.^53^ Our findings suggest that peer problems are notable concerns for junior high school students with ASD. Therefore, interventions focusing on friendships may be needed for adolescents with ASD. In this study, approximately half of the participants were diagnosed with an adjustment disorder. Adjustment disorders are characterized by identifiable psychosocial stressors or life events, such as bullying, school refusal, academic pressure, family discord, abuse, loss experiences, and financial difficulties, that are known to influence SI.^54^, ^55^, ^56^, ^57^ These stressors were not controlled for as potential confounders in the current analysis. Individual stressors should be assessed in future studies to elucidate their specific contributions to SI. The inclusion of such psychosocial variables would enable a more comprehensive understanding of the contextual factors underlying SI in adolescents. Our study also indicated an association between emotional symptoms and SI among junior high school students with ASD. Children with high emotional symptoms, such as irritability, depressive mood, and anxious mood, had higher SI rates during adolescence than those with low symptom levels.^58^ Clinicians should be aware of emotional instability in junior high school students with ASD, particularly during this vulnerable developmental stage.

This study also found that the strength of the autistic traits assessed using the ASQ and SRS‐2 was not associated with the prevalence of SI among junior high school students with ASD. By contrast, Richards et al. (2019) reported that high levels of autistic traits were frequently present in adults with SI and suicide attempts.^59^ Further, Culpin et al. (2018) reported that adolescents with impaired social communication of autistic traits had higher SI risks than those without that trait.^59^, ^60^ These findings differed from our findings. Moreover, Mayes et al. (2013) also reported that autism severity or IQ did not alter SI frequency in adolescents.^61^ Therefore, screening all patients with ASD for SI is important because SI is present across the autism spectrum regardless of autism severity.

This study has some limitations. First, it is a cross‐sectional observational study and therefore cannot indicate cause‐and‐effect relations as longitudinal studies can. Future longitudinal studies are required to support our findings. Second, participants were recruited in a clinical setting, limiting the generalizability of our results to the broader population of junior high school students. Third, we did not control for potential confounding factors, such as domestic environment, including family structure, and economic status. Fourth, SI was assessed using a single item from the GHQ30. Although this item has been used as a proxy for SI in previous studies, the GHQ30 is not specifically designed to evaluate suicide risk. Therefore, the assessment may have been influenced by general psychological distress or depressive symptoms. Future studies should incorporate validated and standardized instruments for suicide risk assessment (e.g., the Columbia Suicide Severity Rating Scale or the Suicidal Ideation Questionnaire) to ensure more reliable and clinically meaningful evaluation. Fifth, of the 470 total participants, 250 were included in the final analysis after applying exclusion criteria. Among the participants, only 84 were individuals with ASD, representing a highly selective subgroup. This small number may limit the generalizability of our findings to the broader ASD population. Furthermore, although sex was included in the regression model, sex‐stratified analyses were not conducted. Future research should explore sex‐specific risk patterns in adolescent SI, particularly within the ASD population, to clarify differential risk profiles and inform targeted interventions.

In conclusion, our findings indicate that approximately 40% of Japanese junior high school students at a child and adolescent psychiatric center, with or without ASD, had SI. Psychiatric clinicians should be cautious about SI in junior high school patients in clinical settings. Furthermore, these results suggest that interventions focusing on improving friendships of junior high school students with ASD may help address SI and suicidal tendencies.

## AUTHOR CONTRIBUTIONS

Yu Matsumoto designed the study, managed data collection, conducted statistical analyses, and prepared the initial manuscript. Kentaro Kawabe and Fumie Horiuchi contributed to the study design, data collection, statistical analysis, and critical revision of the manuscript for important intellectual content. All authors contributed substantially to the interpretation of the clinical data. Mariko Eguchi and Shu‐ichi Ueno provided critical comments that significantly enhanced the final draft of this manuscript. All authors approved the final version of the manuscript.

## CONFLICT OF INTEREST STATEMENT

The authors declare no conflicts of interest.

## ETHICS APPROVAL STATEMENT

The study was approved by the Institutional Review Board of Ehime University Graduate School of Medicine (IRB No. 1607010).

## PATIENT CONSENT STATEMENT

Written informed consent was obtained from all parents and students.

## CLINICAL TRIAL REGISTRATION

N/A.

## Data Availability

The original contributions presented in the study are included in the article/supplementary material, further inquiries can be directed to the corresponding author.
