# Correction to “Factors associated with suicidal ideation in junior high school students with autism spectrum disorder in Japan: A cross‐sectional observational study”

**DOI:** 10.1002/pcn5.70317

**Published:** 2026-03-08

Matsumoto Y, Kawabe K, Horiuchi F, Jogamoto T, Hosokawa R, Nakachi K, et al. Factors associated with suicidal ideation in junior high school students with autism spectrum disorder in Japan: a cross‐sectional observational study. Psychiatry Clin Neurosci Rep. 2026;5(1):e70272.

In the first sentence of the second paragraph of the Introduction, the text “The leading cause of death among teenagers in Japan, with a 7.0% suicide rate.” was incorrect.

This should have read:

“According to the WHO database of causes of death in 2021, suicide is the leading cause of death among teenagers in Japan, with a rate of 7 per 100,000 population.”

We apologize for this error.

